# Chemical niches and ionoregulatory traits: applying ionoregulatory physiology to the conservation management of freshwater fishes

**DOI:** 10.1093/conphys/coab066

**Published:** 2021-09-03

**Authors:** Alex M Zimmer, Greg G Goss, Chris N Glover

**Affiliations:** 1 Department of Biological Sciences, University of Alberta, CW 405, Biological Sciences Bldg., Edmonton, Alberta, T6G 2E9, Canada; 2 Faculty of Science and Technology and Athabasca River Basin Research Institute, Athabasca University, Athabasca, Alberta, T9S 3A3, Canada

**Keywords:** Acid, calcium, salinity, sodium

## Abstract

Alterations in water chemistry can challenge resident fish species. More specifically, chemical changes that disrupt ion balance will negatively affect fish health and impact physiological and ecological performance. However, our understanding of which species and populations are at risk from ionoregulatory disturbances in response to changing freshwater environments is currently unclear. Therefore, we propose a novel framework for incorporating ionoregulatory physiology into conservation management of inland fishes. This framework introduces the concepts of fundamental chemical niche, which is the tolerable range of chemical conditions for a given species based on laboratory experiments, and realized chemical niche, which is the range of chemical conditions in which a species resides based on distribution surveys. By comparing these two niches, populations that may be at risk from ionoregulatory disturbances and thus require additional conservation considerations can be identified. We highlight the potential for commonly measured ionoregulatory traits to predict fundamental and realized chemical niches but caution that some traits may not serve as accurate predictors despite being important for understanding ionoregulatory mechanisms. As a sample application of our framework, the minimum pH distribution (realized niche) and survival limit pH (fundamental niche) of several North American fishes were determined by systematic review and were compared. We demonstrate that ionoregulatory capacity is significantly correlated with a realized niche for many species, highlighting the influence of ionoregulatory physiology on fish distribution patterns along chemical gradients. Our aim is that this framework will stimulate further research in this field and result in a broader integration of physiological data into conservation management decisions for inland waters.

## Teaser

Freshwater fish must maintain the concentration of salts in their blood within a narrow range, but this status is challenged by the prevailing water chemistry and its fluctuations. This paper provides a framework for understanding how fish in nature will respond to forecasted future changes in water chemistry.

## Introduction

Freshwater fish populations are faced with numerous threats, including habitat loss and degradation, overexploitation, introduction of invasive species and climate change (Dudgeon *et al.*, 2006; Arthington *et al.*, 2016; Reid *et al.*, 2019). In addition, trends across the globe demonstrate that the chemical composition of inland waters is changing, with salinization (Cañedo-Argüelles *et al.*, 2013; Dugan *et al.*, 2017), acidification (Dunford *et al.*, 2012; Hasler *et al.*, 2018), calcium loss (Keller *et al.*, 2001; Skjelkvåle *et al.*, 2005; Jeziorski *et al.*, 2008), hypoxia (Jenny *et al.*, 2016) and pollution by metals, organics and other emerging contaminants (Murray *et al.*, 2010; Wood, 2012) being of particular concern. All of these factors contribute to inland fishes being among the most vulnerable of all vertebrate groups, with nearly 24% of species being threatened (IUCN, 2021) and significant reductions in taxonomic, functional and phylogenetic biodiversity occurring worldwide (Su *et al.*, 2021). However, identification of the most sensitive fish species and/or the traits possessed by such species is lacking, a knowledge gap that hinders conservation efforts (Miqueleiz *et al.*, 2020).

Trait-based approaches to conservation management aim to predict species- or population-level responses to environmental change using individual-level characteristics (McGill *et al.*, 2006; Kearney *et al.*, 2010; Chown, 2012; Willis *et al.*, 2015; Glover, 2018). Indeed, some physiological traits have been demonstrated to be important predictors of species/population outcomes, such as temperature tolerance and aerobic scope in sockeye salmon (*Oncorhynchus nerka*) (Eliason *et al.*, 2011; Cooke *et al.*, 2012; Patterson *et al.*, 2016). Such examples, in part, have given rise to the field of conservation physiology (Seebacher and Franklin, 2012; Cooke *et al.*, 2013; Coristine *et al.*, 2014). In this Perspective, we propose a trait-based approach focused on ionoregulatory physiology as a tool for predicting the response of freshwater fishes to changing water chemistry conditions.

Virtually all freshwater fishes regulate concentrations of major ions (Na^+^, Cl^−^, Ca^2+^, K^+^, Mg^2+^, SO_4_^2−^) within a narrow range in the blood plasma. Maintaining ion balance involves a co-ordinated response of many organ systems (gills, gut, kidney) and imposes a significant metabolic cost, although estimates of this cost vary substantially across different studies (Kirschner, 1995; Boeuf and Payan, 2001; Ern *et al.*, 2014; Parker *et al.*, 2020). Furthermore, disruption of ion balance in freshwater fishes can have detrimental effects, culminating in osmotic disturbances and consequent cardiovascular failure in severe cases (Milligan and Wood, 1982; Grosell *et al.*, 2002). Therefore, ion regulation is clearly essential to the fitness of freshwater fishes, yet little attempt has been made to use our understanding of ionoregulatory physiology to predict population-level responses to environmental change. This oversight is particularly concerning considering that mechanisms of ion regulation in freshwater fishes are greatly influenced by water chemistry parameters such as pH (McDonald, 1983a), ion content (Gonzalez *et al.*, 2005; Brauner *et al.*, 2013) and contaminants (Wright, 1995; Wood, 2012; Alsop and Wood, 2013), all of which are affected by both climate change and anthropogenic activities. Indeed, large global variations in freshwater chemistry have recently been highlighted as an important factor to consider in environmental risk assessments for aquatic life (Pinheiro *et al.*, 2021). However, linking ionoregulatory physiology to ecological outcomes is challenging because the capacity to maintain ion balance in response to changes in water chemistry can vary substantially among species (Freda and McDonald, 1988; Kefford *et al.*, 2004) or within populations of the same species (Rahel, 1983; Fraser *et al.*, 2008; Whitehead *et al.*, 2013). Furthermore, species/populations often occur naturally across large gradients of water chemistry conditions in the wild, at both temporal and spatial scales, and thus physiology and ecology of species will also vary over time and distance.

**Figure 1:**
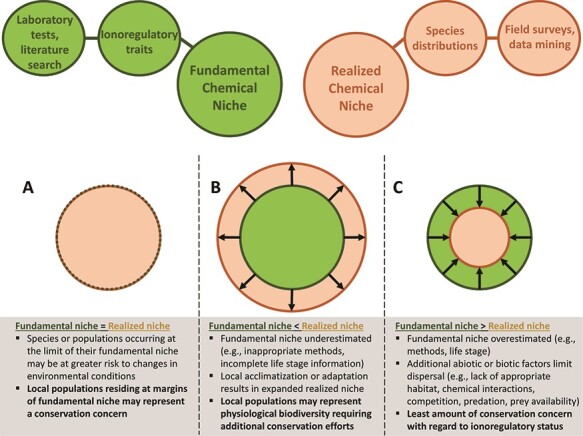
Proposed framework for the integration of ionoregulatory physiology into conservation management of freshwater fishes. The framework relies on determining species-specific fundamental chemical niches, based on ionoregulatory traits assessed through laboratory experiments or literature searches, and realized chemical niches, based on species distribution data. By comparing fundamental and realized chemical niches, conservation managers will be able to arrive at more informed decisions regarding the risk of species or populations of freshwater fishes to future changes in chemical conditions. This niche comparison may also reveal hidden biodiversity in the form of locally adapted populations with broader physiological limitations that may require special considerations for management approaches. Shaded boxes below the niche comparisons (A–C) include bullet points describing factors that might contribute to niche mismatch and the resulting potential implications for conservation management in bolded bullet points.

In this Perspective, we discuss a novel proposal for incorporating ionoregulatory physiology into a framework that can be applied to the conservation management of freshwater fishes. This framework relies on (i) establishing species-specific chemical niches that can act as predictive tools and (ii) identifying ionoregulatory traits that may explain or predict how freshwater fish species will respond to changing water chemistry conditions. Adopting terms from niche theory (Hutchinson, 1957; McGill *et al.*, 2006), we introduce the terms ‘fundamental chemical niche’ (i.e. the tolerable range of chemical conditions for a given species) and ‘realized chemical niche’ (i.e. the range of chemical conditions in which the species resides in nature) and discuss how comparing these niches, and identifying ionoregulatory traits that influence or predict chemical niches, will help inform conservation management decisions (Fig. 1).

## Chemical niches

To predict whether a species or population may be at risk from changes in chemical conditions, it is necessary to understand both its physiological limitations within relevant ranges of chemical conditions (fundamental niche) and how these limitations compare to its natural distribution along chemical gradients in the wild (realized niche). For simplicity, our proposal addresses chemical niches as single variables (e.g. salinity niches, pH niches). Still, we recognize that, in reality, some of these variables co-vary in nature (e.g. ion content and pH) and also have interactive effects on ionoregulatory physiology (e.g. Ca^2+^ and pH). Fundamental chemical niches of freshwater fishes are determined in laboratory settings, resulting from studies examining the physiological limits of different species to characterize traits such as salinity tolerance (Dunson *et al.*, 1993; Ostrand and Wilde, 2001; Kefford *et al.*, 2004) or pH tolerance (Dunson *et al.*, 1977, 1993; Gonzalez and Dunson, 1987, 1989a; Jellyman and Harding, 2014). Realized chemical niches are based on field distribution studies, which to date have examined fish distributions with respect to dissolved oxygen, salinity and pH (Rahel and Magnuson, 1983; Davenport and Sayer, 1993; Graham, 1993; Tremblay and Richard, 1993; Jackson *et al.*, 2001; Ostrand and Wilde, 2001; Kefford *et al.*, 2004).

In niche theory, the realized niche is considered a subset of the fundamental niche such that the fundamental niche is usually greater than the realized niche (Hutchinson, 1957; Soberón and Arroyo-Peña, 2017). In our framework, we acknowledge that a myriad of abiotic and biotic factors, which may or may not be related to ionoregulatory physiology, contribute to differences between fundamental and realized chemical niches (Fig. 1) and that teasing these factors apart is challenging. Indeed, comparison of the temperature niches of two temperate perch species native to Australia failed to demonstrate a relationship between fundamental niche (measured as swimming performance and aerobic capacity) and realized niche (temperature distribution), suggesting that other biotic or abiotic factors contributed to realized niche (Allen-Ankins and Stoffels, 2017). In contrast to this approach, our framework does not suggest that fundamental niche should predict realized niche, due to the many factors that might influence fish distributions, but rather that comparison of these niches can shed light on potential conservation concerns.

One of the abiotic influences on fundamental and realized niches that must be considered is water chemistry itself, with Ca^2+^ concentration being a particularly important modulator of ionoregulatory physiology. The capacity of fishes to maintain ion balance in response to low pH conditions or reductions in ionic strength is influenced by ambient Ca^2+^ concentrations (McDonald *et al.*, 1980, 1983; McDonald, 1983b; McDonald and Rogano, 1986; Gonzalez and Dunson, 1989b; Val *et al.*, 1998; Gonzalez and Preest, 1999) because Ca^2+^ is an integral component of tight junctions that contribute to gill permeability (Hunn, 1985). Realized niches can additionally be influenced by abiotic and biotic factors such as lake area/depth, habitat suitability, temperature, predator/prey interactions, competition or dispersal limitations (Jackson *et al.*, 2001). Therefore, it is possible that differences in ionoregulatory capacity (i.e. fundamental chemical niche) have a negligible influence on species distributions when other abiotic/biotic factors have a greater impact. Nevertheless, our proposed framework aims to serve as a predictive tool for identifying instances where disruptions in ion balance may pose ecological risks for specific fish populations (Fig. 1).

Comparing fundamental and realized niches on species- and site-specific bases will allow conservation managers to identify populations that may be at risk from ionoregulatory disturbances. First, populations of fishes residing at the margins of their fundamental niche (i.e. fundamental niche = realized niche; Fig. 1A) are potentially at risk from deviations in chemical conditions. For instance, populations existing at the lower threshold of their fundamental salinity niche (i.e. inhabiting dilute soft waters) may be at particular risk to declines in Ca^2+^. Reductions in Ca^2+^ concentration have been observed in many regions (Keller *et al.*, 2001; Skjelkvåle *et al.*, 2005; Jeziorski *et al.*, 2008) and will affect ionoregulatory capacity through the known importance of Ca^2+^ in the acclimation of fish to low ionic strength conditions (McDonald and Rogano, 1986). However, lower salinity thresholds are seldom measured in the laboratory, and this fundamental niche is currently poorly defined for most freshwater fishes. At the other extreme, some fishes inhabiting natural inland saline lakes may reside near the extent of their upper salinity tolerance, yet these environments are currently threatened by increasing salinity, driven by climate change and anthropogenic activities (Covich *et al.*, 1997; Williams, 2002). There are reported instances where increases in salinity of these environments, attributed to diversion of freshwater inputs, have already been correlated to decreases in fish biodiversity (Williams, 2002). Therefore, in these examples, establishing fundamental salinity niches (both upper and lower salinity tolerance) is an important step towards identifying species and populations that may be at risk.

Second, fishes found outside their fundamental chemical niche (i.e. fundamental niche < realized niche; Fig. 1B) also represent a case where increased conservation efforts may be needed. In such scenarios, identified populations may have become locally adapted, residing outside the expected range of tolerable chemical conditions based on physiological limits determined in laboratory experiments, and therefore represent physiological biodiversity. For example, Atlantic salmon (*Salmo salar*) alevins of parents originating from a naturally acidic river (Tusket River, Nova Scotia, Canada; pH = 4.6–5.2) had higher survivorship under acidic rearing conditions than alevins of parents from non-acidic sites or a commercial farm (Fraser *et al.*, 2008). This finding, where the realized chemical niche of this population (Tusket River) exceeded the fundamental chemical niche based on experiments using farmed fish, was attributed to local adaptation. Interestingly, interbreeding between acid-adapted and non-acid-adapted salmon, which might occur when farmed salmon escape from aquaculture settings, resulted in a decreased acid tolerance in the F1 generation, but not in the F2 generation (Fraser *et al.*, 2008). Indeed, protecting locally adapted populations and their physiological diversity from introgression with non-adapted species is an important conservation concern (Rhymer and Simberloff, 1996; Bohling, 2016).

Finally, populations of fishes found within the extent of their fundamental chemical niche (fundamental niche > realized niche; Fig. 1C) are the groups of least conservation concern, at least in terms of ionoregulatory status. Under this scenario, there exists a buffer of physiological capacity against changes in environmental conditions. Notably, in all cases, it is important to consider whether the fundamental chemical niche was assessed using relevant water chemistry conditions, appropriate methods (e.g. abrupt versus gradual salinity acclimation; Kefford *et al.*, 2004) or at appropriate life stages (e.g. DeLonay *et al.*, 1993; Whiterod and Walker, 2006) before making conclusions regarding conservation risks.

## Ionoregulatory traits

**Figure 2:**
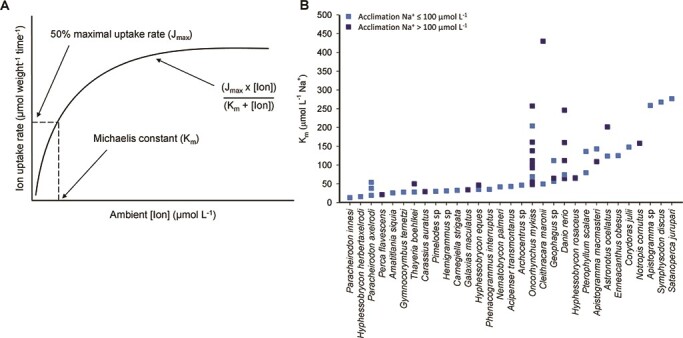
(A) Michaelis–Menten relationship between ambient ion concentration and ion uptake rate. The Michaelis affinity constant (K_m_) is the ambient ion concentration at which 50% maximal uptake rate (J_max_) occurs. Relationship of the line is defined by the equation: Ion uptake rate = (J_max_ x [Ion])/(K_m_ + [Ion]). (B) Plot of K_m_ values for Na^+^ uptake measured in freshwater fish species acclimated to [Na^+^] > 100 μmol l^−1^ (dark blue squares) or [Na^+^] ≤ 100 μmol l^−1^ (light blue squares); 100 μmol l^−1^ Na^+^ is often used as a ‘low’ acclimation condition in the literature and was the median acclimation [Na^+^] across the studies included in the plot. Species are listed in order of ascending K_m_ values. (Data obtained from Lauren and Mcdonald, 1987; Freda and Mcdonald, 1988; Gonzalez and Dunson, 1989a; Goss and Wood, 1990; Postlethwaite and McDonald, 1995; Gonzalez *et al.*, 2017, 2018, 2021; Morgan *et al.*, 1997; Gonzalez *et al.*, 1997, 2002; Salama *et al.*, 1999; Gonzalez and Preest, 1999; Gonzalez and Wilson, 2001; Grosell and Wood, 2002; Boisen *et al.*, 2003; Matsuo *et al.*, 2004; Preest *et al.*, 2005; Matsuo and Val, 2007; Kumai *et al.*, 2011; Glover *et al.*, 2012; Duarte *et al.*, 2013; Al-Reasi *et al.*, 2016; Shartau *et al.*, 2017; see Table S1 for further details.)

The fundamental chemical niche is ultimately a product of organismal physiology, described as a filter between environmental conditions and ecological success (Seebacher and Franklin, 2012). Consequently, the physiological traits underlying ionoregulatory performance are likely to be important in predicting how freshwater fish populations will respond to changes in water chemistry. Here, we define an ionoregulatory trait as any biological characteristic that contributes to, or explains variations in, ionoregulatory performance. Basic ionoregulatory traits such as ion fluxes (Giacomin *et al.*, 2020), blood/tissue ion content (Blanchard and Grosell, 2006), transepithelial potential (Wood *et al.*, 2020), stress indicators (e.g. cortisol/glucose responses; Kammerer *et al.*, 2010), behaviour (DeLonay *et al.*, 1993; Ikuta *et al.*, 2003), metabolic rate/metabolic status (Parker *et al.*, 2020) or general fitness traits (i.e. mortality, growth, development, reproduction) can underlie more complex traits like substrate affinity for ion uptake (e.g. Goss and Wood, 1990; Gonzalez *et al.*, 2002; Boisen *et al.*, 2003; Fig. 2), salinity/salt tolerance (e.g. Ostrand and Wilde, 2001; Kefford *et al.*, 2004; Wood *et al.*, 2020), pH tolerance (e.g. Freda and Mcdonald, 1988; Gonzalez and Dunson, 1989a; Wilkie and Wood, 1996), hypoxia tolerance (e.g. Wood *et al.*, 2007; Iftikar *et al.*, 2010; Giacomin *et al.*, 2020), temperature tolerance (e.g. Gonçalves *et al.*, 2006) or trace metal tolerance (e.g. Grosell *et al.*, 2002). Notably, some of these ionoregulatory traits, such as ion substrate transport affinity, salinity tolerance and pH tolerance, directly reflect ionoregulatory function. Conversely, others are characteristics that are likely more dependent on other physiological systems (e.g. cardiovascular physiology in hypoxia/temperature tolerance, detoxification pathways in metal tolerance) but which have an important ionoregulatory component. For example, in the hypoxia-tolerant Amazonian oscar (*Astronotus ocellatus*) and mummichog (*Fundulus heteroclitus*), depression of ion flux rates is a key adaptation in minimizing metabolic rate when oxygen availability is low (Wood *et al.*, 2007; Giacomin *et al.*, 2020).

Although the contribution of various ionoregulatory traits to overall ionoregulatory performance has been well described in physiological studies, their predictive capacity for defining chemical niches is less clear. Therefore, an important step in our proposed framework is to test whether a given ionoregulatory trait, or set of traits, is predictive of fundamental chemical niches using laboratory tests and to address if and why the trait was predictive of realized niches using distribution surveys. Previous work has, in fact, already demonstrated that some ionoregulatory traits may be predictive of chemical niche.

For example, in two closely related sunfish species, *Enneacanthus obsesus* and *Enneacanthus gloriosus*, pH tolerance assessed in the laboratory was predictive of distribution (Gonzalez and Dunson, 1991). While both species are considered acid tolerant, *E. obesus* has a higher tolerance than *E. gloriosus*. When the more sensitive species was exposed to pH 4, whole-body Na^+^ content was significantly reduced after 1 week and growth rate was inhibited after 12 weeks. In contrast, no effects were observed following the same acid exposure in the more tolerant species (Gonzalez and Dunson, 1987, 1989a). These relative tolerance patterns observed in the laboratory reflect natural distributions, with *E. gloriosus* being excluded from the most acidic waters of the natural range of *E. obesus* (Gonzalez and Dunson, 1991). Similarly, in yellow perch (*Perca flavescens*) and Atlantic salmon (*S. salar*), pH tolerance was higher in individuals of acidic water origin (pH 4–5) compared to those sourced from neutral environments (pH 7–8) (Rahel, 1983; Fraser *et al.*, 2008).

Salinity tolerance measured in the laboratory can also be predictive of species distribution in the wild. For example, a strong correlation was found between experimental salinity tolerance (usually measured as the salinity concentration lethal to 50% of individuals; LC_50_) and the maximum salinity at which the species occurred in the field (maximum field distribution) for a number of freshwater fish species native to southeastern Australia (Kefford *et al.*, 2004). Notably, the method of assessing salinity LC_50_ (direct transfer or slow acclimation) resulted in different correlations, with slow salinity acclimation being more predictive of maximum field distribution (Kefford *et al.*, 2004). Salinity tolerance was also suggested to influence fish assemblages in streambed pools of the Brazos River Basin (TX, USA) that become saline due to evaporation (Ostrand and Wilde, 2001).

Ionoregulatory physiology also underpins the sensitivity of freshwater fishes to some trace metal pollutants, and disruption of Na^+^ balance in particular has been proposed as a lethal mechanism of action in response to exposure to a variety of pollutants (Grippo and Dunson, 1991; Alsop and Wood, 2013). Copper (Cu^2+^) and silver (Ag^+^), for example, are capable of mimicking Na^+^, gaining entry into a fish via Na^+^ uptake pathways, and thereafter disrupting Na^+^ balance through inhibition of the basolateral sodium pump that drives Na^+^ uptake (Bury and Wood, 1999; Grosell and Wood, 2002; Goss *et al.*, 2011). This results in a scenario whereby individuals with higher Na^+^ turnover rates generally exhibit a greater risk for Cu^2+^/Ag^+^ accumulation and toxicity (Grosell *et al.*, 2002; Harley and Glover, 2014). This physiological mechanism has been critically important in the development of predictive models for identifying fish species at greatest risk of toxicity from the presence of Cu^2+^ and Ag^+^ in freshwaters, forming part of the basis of regulatory decision-making tools for establishing water quality criteria for different metals (Paquin *et al.*, 2002). Incorporating physiological data into these models/tools is a clear example of how ionoregulatory traits can be applied to conservation management for inland waters. Importantly, such models must account for multiple water chemistry parameters, as demonstrated by the case of aluminium (Al). This trace metal is an ionoregulatory toxicant (Goss and Wood, 1988; Wood *et al.*, 1990) that is mobilized and becomes more soluble at low pH (Nelson and Campbell, 1991; Gensemer and Playle, 1999), highlighting the complexity of predicting population responses to multiple simultaneous alterations in water chemistry conditions.

On the other hand, some ionoregulatory traits that have contributed to our mechanistic understanding of ionoregulatory physiology may not necessarily be useful predictors of chemical niche. Substrate affinity for ion uptake, for instance, has been used to understand mechanisms of ion acquisition. Rates of ion absorption/influx/uptake in freshwater fishes are modelled by Michaelis–Menten kinetics (Fig. 2A), whereby ion uptake rate is a function of ambient ion concentration. Substrate affinity is defined by the Michaelis affinity constant (K_m_), which differs across species and acclimation conditions such as environmental ion concentration (Fig. 2B). If this ionoregulatory trait were an important determinant of chemical niche, we would predict that fishes native to conditions that are Na^+^-deficient, for example, should have a low K_m_ value (i.e. high affinity) for Na^+^ uptake. This is true for the characiform fishes of the acidic and ion-poor Rio Negro in the Brazilian Amazon (*Gymnocorymbus ternetzi*, *Hemigrammus* sp., *Hyphessobrycon* sp., *Nematobrycon palmeri*, *Paracheirodon* sp., *Thayeria boehlkei*) that have a high affinity (K_m_ < 50 μmol L^−1^) Na^+^ uptake system that matches their Na^+^-deficient environment (Na^+^ = 16.5 μmol L^−1^; Gonzalez *et al.*, 2005), but not true of the cichlid species *Symphysodon discus* and *Satanoperca jurupari*, also native to the Rio Negro (Fig. 2B). These cichlid species appear to utilize a different ionoregulatory strategy, one that minimizes rates of Na^+^ loss (Gonzalez *et al.*, 2002, 2005; Duarte *et al.*, 2013; Morris *et al.*, 2021), thereby maintaining ion balance even when Na^+^ affinity does not match prevailing ionic conditions. Notably, it is not unusual that seemingly important traits fail to predict ecological performance or species distribution in relevant environmental gradients (e.g. upper thermal tolerance; Sunday *et al.*, 2012; Cahill *et al.*, 2013; Evans *et al.*, 2015). Consequently, it is important to employ a broad assessment of ionoregulatory traits to determine which are likely to be useful for predicting or understanding chemical niches.

## Applying the framework

**Figure 3:**
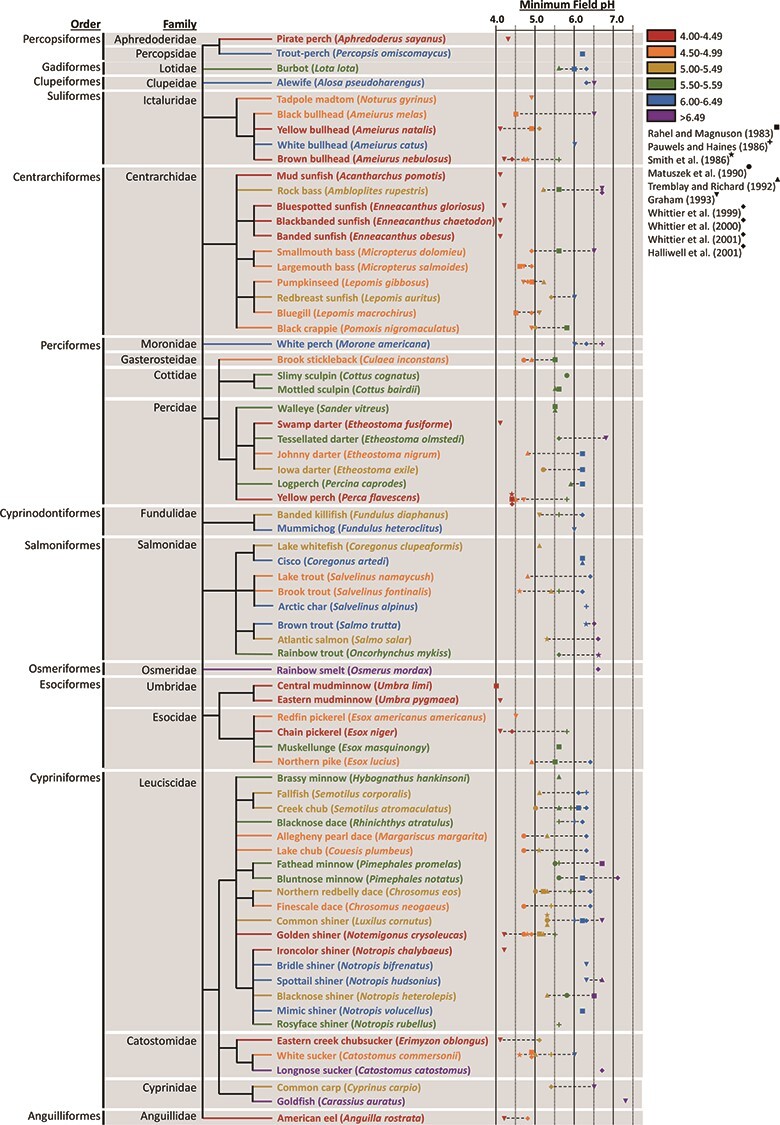
Minimum field pH of 72 freshwater fish species in over 1000 lakes surveyed in different geographic regions of North America in 7 studies. Species are arranged by phylogeny constructed using the NCBI Taxonomy Browser and Phylogeny.fr (Dereeper *et al.*, 2008); text colour refers to the lowest minimum field pH reported for that species according to the legend in the figure. Symbols represent the study from which the minimum field pH data was obtained; see legend for details. Note that the study represented by diamonds consists of four different publications addressing different fishes in the same study lakes. (Data obtained from Rahel and Magnuson, 1983; Pauwels and Haines, 1986; Smith *et al*., 1986; Matuszek *et al*., 1990; Graham, 1993; Tremblay and Richard, 1993; Whittier *et al*., 1999, 2000, 2001; Halliwell *et al*., 2001.)

**Figure 4:**
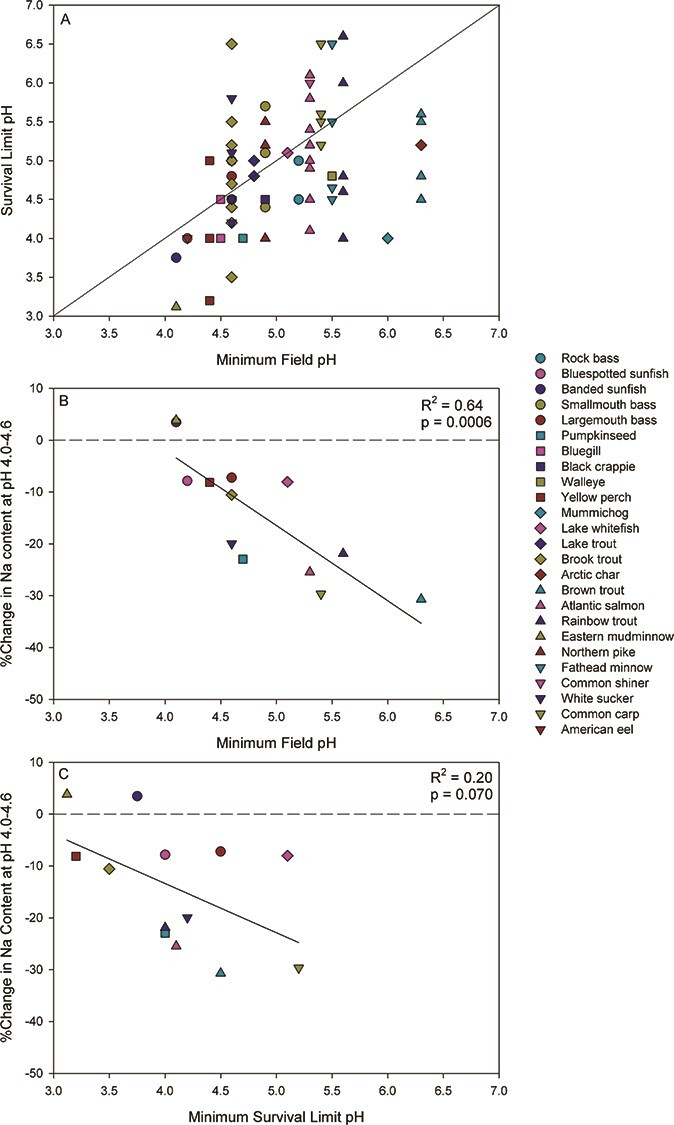
(A) Relationship between lowest minimum field pH and survival limit pH for 25 of the species included in Fig. 3. Relationships between % change in Na content at pH 4.0–4.6 and (B) minimum field pH and (C) minimum survival limit pH for 13 of the species included in Fig. 3. Species are represented as different symbol and colour combinations according to the figure legend. Survival limit data obtained from: rock bass (McCormick *et al*., 1989; Eaton *et al*., 1992); bluespotted sunfish (Gonzalez and Dunson, 1989a); banded sunfish (Gonzalez and Dunson, 1989a); smallmouth bass (Kwain *et al.*, 1984; Kane and Rabeni, 1987; Holtze and Huchinson, 1989); largemouth bass (Orsatti and Colgan, 1987; McCormick *et al*., 1989; Eaton *et al*., 1992; McCormick and Jensen, 1992); pumpkinseed (Fraser and Harvey, 1984); bluegill (Ellgaard and Gilmore III, 1984; Palmer et al., 1988); black crappie (McCormick *et al*., 1989); walleye (Holtze and Huchinson, 1989); yellow perch (Rahel, 1983; Freda and McDonald, 1988; McCormick *et al*., 1989; Eaton *et al*., 1992); Mummichog (Gonzalez *et al*., 1989); lake whitefish (Holtze and Huchinson, 1989); lake trout (Gunn and Noakes, 1987; Hutchinson *et al*., 1989); brook trout (Daye and Garside, 1975; Menendez, 1976; Trojnar, 1977a; Cleveland *et al*., 1986; Hunn *et al*., 1987; Hurley *et al*., 1989; Hutchinson *et al*., 1989; Ingersoll *et al*., 1990a, 1990b; Wood *et al*., 1990); Arctic char (Jagoe *et al*., 1984); brown trout (Carrick, 1979; Brown, 1983; Reader *et al*., 1989; Sayer *et al*., 1989, 1991); Atlantic salmon (Daye and Garside, 1977, 1979; Peterson *et al*., 1980; Lacroix *et al*., 1985; Farmer *et al*., 1989; Fivelstad *et al*., 2004; Fraser *et al*., 2008; Kroglund *et al*., 2008); rainbow trout (Kwain, 1975; Neville, 1979; Graham and Wood, 1981; Nelson, 1982; Audet *et al*., 1988; Thomsen *et al*., 1988; Balm and Pottinger, 1993); eastern mudminnow (Dederen *et al*., 1986); northern pike (Johansson and Kihlström, 1975; Vuorinen *et al*., 1993; Duis and Oberemm, 2000; Keinänen *et al*., 2000, 2004); fathead minnow (Mount, 1973; Palmer *et al*., 1988, 1989; Grippo and Dunson, 1991); common shiner (Holtze and Huchinson, 1989); white sucker (Beamish, 1972; Trojnar, 1977b; Fraser and Harvey, 1984; Holtze and Huchinson, 1989); common carp (Korwin-Kossakowski, 1988; Oyen *et al*., 1991; Stouthart *et al*., 1994; Sapkale *et al*., 2011); American eel (Reynolds, 2011). Sodium content data obtained from: Dively *et al*., 1977; Neville, 1979; McDonald *et al*., 1980; McDonald and Wood, 1981; Ultsch *et al*., 1981; Rahel, 1983; McDonald, 1983b; Fraser and Harvey, 1984; Giles *et al*., 1984; Höbe *et al*., 1984; Canfield *et al*., 1985; Stuart and Morris, 1985; Scherer, 1986; Gonzalez and Dunson, 1987, 1989a; Booth *et al*., 1988; Wood *et al*., 1988; Reader *et al*., 1989; Farmer *et al*., 1989; Wendelaar Bonga *et al*., 1990; Sayer *et al*., 1991; Ye *et al*., 1991; Wright *et al*., 2014.

To demonstrate how ionoregulatory traits can predict realized chemical niche, thereby highlighting the utility of our framework, we compared the realized and fundamental pH niches of several freshwater fish species in North America and related these niches to changes in Na^+^ content. First, we summarized the realized pH niches (minimum field pH) of 72 inland fish species in over 1000 lakes surveyed across several geographic regions of Canada and the USA (Fig. 3). In this figure, the species are arranged by phylogeny to highlight notable trends such as the apparent acid-tolerant nature of centrarchid fishes and the general acid sensitivity of fishes in the genus *Notropis*, with the exception of the ironcolor shiner (*Notropis chalybaeus*). These phylogenetic relationships may prove useful for broadly determining which species may be at risk from anthropogenic acidification or other chemical disturbances and for identifying species that may serve as representative models in future research. The common shiner (*Luxilus cornutus*) (Figs 3, 4A), for instance, has been used as a representative acid-sensitive species in previous comparative physiology research (Freda and McDonald, 1988; McDonald *et al.*, 1991).

Second, we performed a systematic literature review to determine the fundamental pH niche (survival limit pH) for as many species in Fig. 3 as possible. We considered the survival limit pH as the lowest pH in a given study that resulted in ≤20% mortality and restricted our search to studies that exposed fish for at least 24 h to avoid acutely toxic effects of H^+^. We did not control for life stage or water chemistry, except for the omission of experiments that co-exposed fish to low pH and trace metals (e.g. Al). Survival limit pH was determined for 25 species from Fig. 3. For many species, survival limit pH varied substantially across studies, which was likely a result of differences in water chemistry (e.g. Ca^2+^) and life stage. Survival limit pH also showed no apparent relationship with minimum field pH (Fig. 4A). However, based on our framework, we would predict that species with data points falling along the line of conformity between minimum field pH and survival limit pH (Fig. 4A) have a fundamental pH niche equal to the realized pH niche (Fig. 1A) and may represent a concern for conservation management. Moreover, those species with data points above the line of conformity have a fundamental pH niche that is less than the realized pH niche (Fig. 1B). In these cases, where fishes reside in waters with a pH that has been demonstrated to be toxic in survival studies, it is possible that differences in water chemistry (e.g. ionic strength, Ca^2+^) or life stage between field sites and laboratory studies account for the mismatch between niches; however, these may also be cases of local acid adaptation/acclimation. We believe that this type of comparative analysis is the first step for identifying populations where further attention may be needed in terms of assessing ionoregulatory status and/or deciding upon conservation intervention.

Third, to determine the extent to which ionoregulatory physiology influences pH niches, an additional literature review was conducted to determine species-specific responses of plasma and/or whole-body Na levels to low pH exposure. This search was again limited to experiments of at least 24 h to avoid acute effects and further limited to studies that exposed fish to pH 4.0–4.6 because this pH level was generally the lower threshold of fundamental and realized niches of the most acid-tolerant species in our study (Figs 3, 4). Water chemistry was again not accounted for, except to exclude studies with trace metal co-exposure. The difference in plasma and/or whole-body Na content between fish exposed to pH 4.0–4.6 and fish exposed to control conditions (pH 6.5–8) was calculated as ‘%Change in Na content at pH 4.0–4.6’. This metric therefore represents ionoregulatory pH tolerance, whereby species with a lower value are considered more acid tolerant. A significant correlation (R^2^ = 0.64; *P* = 0.0006) was found between minimum field pH and ionoregulatory pH tolerance (Fig. 4B), clearly demonstrating that this ionoregulatory trait is characteristic of realized pH niche and that greater ionoregulatory pH tolerance imparts a broader realized pH niche. Furthermore, based on this relationship, we would predict that species at risk from acid stress would display lower Na^+^ content relative to individuals in circumneutral waters. Interestingly, however, there was no significant relationship between ionoregulatory pH tolerance and minimum survival limit pH (i.e. fundamental pH niche) (Fig. 4C), indicating that ionoregulatory disturbances may not always be the lethal mechanism of action in low pH exposure. In addition to disruptions in ion balance, acid exposure may also result in acid-based dysregulation, respiratory disturbance and/or gill damage, which might contribute to lethality, depending on water chemistry conditions and species (McDonald, 1983a).

Overall, this systematic review highlights that ionoregulatory traits can influence the distribution of fishes along chemical gradients and that, at least for pH tolerance, the simple measurement of plasma or whole-body Na^+^ content may be a useful metric for assessing whether individuals in a given environment are experiencing ionoregulatory disturbances and may therefore be at risk from perturbations in water chemistry conditions. In an *in situ* caged bioassay study of brook trout in episodically acidified streams in Great Smoky Mountains National Park, USA, whole-body Na^+^ content was correlated with the natural differences in stream pH and Al concentration that occurred during the pulse episodes (Neff *et al.*, 2009), further highlighting the applicability of this metric to conservation monitoring. Notably, however, a reduction in plasma Na may actually underlie the physiological acclimation response to low pH in some species (Audet *et al.*, 1988; Gonzalez and Dunson, 1989a), complicating the applicability of this parameter. However, it is not known whether this reduction in Na content leaves these fishes more vulnerable to other environmental stressors (e.g. hypoxia, pollutants).

## Limitations, perspectives and future directions

Ionoregulatory physiology appears to play a key role in determining the success or failure of inland fishes inhabiting chemically altered environments. Our proposed framework serves as a foundation for identifying situations of concern with regard to risk from ionoregulatory disturbances and provides a basis for building a broader understanding of the ecophysiological implications of ionoregulatory traits of freshwater fishes. By comparing the fundamental and realized chemical niches of inland fishes, conservation managers can identify populations that may be at risk from future environmental change, such that individuals of these populations can be assessed for ionoregulatory disturbances using simple metrics such as ion content measurements. In addition, through this framework, cases can be identified where physiological data regarding ionoregulatory traits and fundamental chemical niches are lacking, thereby informing priorities for conservation/ionoregulatory physiology research.

It is important to note that several limitations to this framework currently exist. First, there is a lack of data regarding both fundamental and realized chemical niches for most freshwater fishes, particularly concerning potential shifts in niche over life history. The speciosity of freshwater fishes clearly represents a research challenge; however, a phylogenetic approach (Fig. 3) may allow researchers to identify broad trends for conservation management purposes and identify key species to act as representative models for laboratory research. Second, it is currently unclear which basic traits (e.g. ion content, metabolic rate, growth/body size) may serve as important indicators of potential niche mismatching (Figs 1A, B) for different chemical niches. While Na^+^ content may be a reliable indicator for acid stress (Fig. 4B), it has its limitations and may not be a relevant indicator for other chemical niches. Third, most studies have examined the effects of altered water chemistry as single variables, but chemical niches will likely need to be multivariate given the covariance of many water chemistry parameters in nature and their interactive effects on ionoregulatory physiology. It is possible, however, to design multivariate studies tailored to address emerging environmental issues (e.g. interactions of Al, pH, and Ca^2+^; Ingersoll *et al.*, 1990b; Wood *et al.*, 1990). Lastly, although controversial (Pulliam, 2000), niches are generally theorized as having dimensions, such as bell-shaped distributions (Hutchinson, 1957). Consequently, physiological/ecological performance is predicted to be optimal at a particular point along an environmental gradient. Presently, we have left the shape of chemical niches undefined (i.e. circles in Fig. 1) because chemical optima for ionoregulatory performance in fishes are still debated in comparative physiology (e.g. the salinity at which ionoregulatory costs are lowest; Ern *et al.*, 2014). Therefore, determining these optima and establishing dimensionality for chemical niches remains a challenge for future applications of this niche framework.

There are a number of pressing environmental issues for which our proposed framework can be adopted to better understand and predict the fate of freshwater fishes. Ionoregulatory physiology has proven to be pivotal for understanding individual-level responses to emerging concerns such as salinization, calcium decline, acidification, deoxygenation and climate change (McDonald *et al.*, 1980; McDonald, 1983a; Gonçalves *et al.*, 2006; Iftikar *et al.*, 2010; Wood *et al.*, 2020), and incorporation of ionoregulatory traits into conservation efforts is therefore a critical step towards well-informed management decision-making. Moreover, given the general sentiment that environmental change tends to favour invasive species (Chown, 2012), primarily due to shifts in environmental conditions away from the optima of indigenous species adapted to prevailing conditions, a better understanding of the chemical niches of invasive species might contribute to forecasting invasion potential. For example, ecological niche-based modelling of the invasion potential of common carp (*Cyprinus carpio*) identified nine variables that predicted the presence/abundance of carp in lakes in MN and ND, USA, one of which was alkalinity, accounting for up to 15% of the predictive power of the model (Kulhanek *et al.*, 2011). This finding is in general agreement with our analysis that identified carp as only moderately acid tolerant (fundamental/realized niche = pH 5.0–5.5; Figs 3, 4), and thus implies that the invasive potential of carp may be affected by ionoregulatory pH tolerance.

Overall, our goal is that this framework will act as a catalyst for directing new avenues of research and serve as a starting point for broader integration of physiological data into conservation management decisions for inland waters, similar to what has occurred with the establishment of water quality criteria for metal toxicants (Paquin *et al.*, 2002).

## Supplementary material

Supplementary material is available at *Conservation Physiology* online.

## Funding

This work was supported by the Natural Sciences and Engineering Research Council of Canada Discovery Grants to C.N.G. [# 04314] and G.G.G. [#203736]. C.N.G. is supported by a Campus Alberta Innovates Program research chair.

## Supplementary Material

Table_S1_coab066
