# Supplementary material for: Chemical niches and ionoregulatory traits: applying ionoregulatory physiology to the conservation management of freshwater fishes
Source: Conserv Physiol. 2021 Sep 3;9(1):coab066. doi: 10.1093/conphys/coab066 (PMC8415428; doi:10.1093/conphys/coab066)
Supplement: Table_S1_coab066 [file table_s1_coab066.docx]

Table S1. Michaelis constant (K_m_; µmol L^-1^) and maximal uptake rate (J_max_; µmol kg^-1^ h^-1^) for Na^+^ uptake in several species, mass (g) of the individuals used in the study, and acclimation conditions used in the study. Species appear in the same order as presented in Figure 2.

|  |  |  |  |  | **Acclimation Conditions** | | | | |  |
| --- | --- | --- | --- | --- | --- | --- | --- | --- | --- | --- |
| **Species** | **Common Name** | **Km**  **(µmol L^-1^)** | **Jmax**  **(µmol kg^-1^ h^-1^)** | **Mass (g)** | **[Na] (mmol L^-1^)** | **[Cl] (mmol L^-1^)** | **[Ca] (mmol L^-1^)** | **pH** | **Temp (°C)** | **Reference** |
| *Paracheirodon innesi* | Neon tetra | 12.9 | 448 | 0.347 | 0.05 | 0.2 | 0.05 | 6.5 | 26 | (Gonzalez and Preest, 1999) |
| *Hyphessobrycon herbertaxelrodi* | Black neon tetra | 15.7 | 1033 | 0.498 | 0.1 | 0.3 | 0.1 | 7.5 | 26 | (Gonzalez *et al.*, 2017) |
| *Paracheirodon axelrodi* | Cardinal tetra | 19 | 2076 | 0.063 | 0.036 | 0.028 | 0.012 | 6.35 | 28 | (Matsuo and Val, 2007) |
| *Paracheirodon axelrodi* | Cardinal tetra | 38 | 2279 | 0.063 | 0.052 | 0.028 | 0.012 | 3.72 | 28 | (Matsuo and Val, 2007) |
| *Paracheirodon axelrodi* | Cardinal tetra | 53.7 | 773 | 0.545 | 0.05 | 0.2 | 0.05 | 6.5 | 26 | (Gonzalez and Wilson, 2001) |
| *Perca flavescens* | Yellow perch | 21 | 249 | 10 | 0.11 | 0.13 | 0.065 | 6.5 | 15 | (Freda and McDonald, 1988) |
| *Amatitlania siquia* | Red point cichlid | 25.6 | 647.2 | 1.244 | 0.1 | 0.3 | 0.1 | 7.5 | 26 | (Gonzalez *et al.*, 2017) |
| *Gymnocorymbus ternetzi* | Black widow tetra | 27.7 | 691.3 | 1.5 | 0.05 | 2 | 0.05 | 6.5 | 26 | (Gonzalez *et al.*, 1997) |
| *Thayeria boehlkei* | Penguin tetra | 28 | 1468 | 0.512 | 0.1 | 0.3 | 0.1 | 7.5 | 26 | (Gonzalez *et al.*, 2018) |
| *Thayeria boehlkei* | Penguin tetra | 49.9 | 1473 | 0.512 | 1 | 1.2 | 0.1 | 7.5 | 26 | (Gonzalez *et al.*, 2018) |
| *Carassius auratus* | Goldfish | 28.9 | 497.3 | NA | 0.3 | 1.2 | 0.3 | 6.5 | 26 | (Preest *et al.*, 2005) |
| *Pimelodes* sp | Pimelodes | 29.7 | 1263.9 | 1.67 | 0.0296 | 0.0228 | 0.0094 | 6 | 25 | (Gonzalez *et al.*, 2002) |
| *Hemigrammus* sp | Hemmigramus | 30.9 | 1440 | 1.19 | 0.0188 | 0.0214 | 0.0088 | 6.5 | 25 | (Gonzalez *et al.*, 2002) |
| *Carnegiella strigata* | Marbled hatchetfish | 32.5 | 1225 | 1.64 | 0.0188 | 0.0214 | 0.0088 | 6.5 | 25 | (Gonzalez *et al.*, 2002) |
| *Galaxias maculatus* | Inanga | 34 | 126 | 1.34 | 0.32 | NA | NA | 7 | 15 | (Glover *et al.*, 2012) |
| *Hyphessobrycon eques* | Serpae tetra | 34.8 | 1111 | 1.039 | 0.1 | 0.3 | 0.1 | 7.5 | 26 | (Gonzalez *et al.*, 2018) |
| *Hyphessobrycon eques* | Serpae tetra | 46.4 | 1144 | 1.039 | 1 | 1.2 | 0.1 | 7.5 | 26 | (Gonzalez *et al.*, 2018) |
| *Phenacogrammus interruptus* | Congo tetra | 34.9 | 1673 | 0.862 | 0.1 | 0.3 | 0.1 | 7.5 | 26 | (Gonzalez *et al.*, 2017) |
| *Nematobrycon palmeri* | Emperor tetra | 41.7 | 1342 | 0.327 | 0.1 | 0.3 | 0.1 | 7.5 | 26 | (Gonzalez *et al.*, 2018) |
| *Acipenser transmontanus* | White sturgeon | 43 | 204 | 2.44 | 0.07 | 0.073 | 0.089 | 6.3 | 15 | (Shartau *et al.*, 2017) |
| *Archocentrus* sp | Archocentrus | 46.2 | 339.9 | 1.8 | 0.1 | 0.3 | 0.1 | 7.5 | 26 | (Gonzalez *et al.*, 2017) |
| *Oncorhynchus mykiss* | Rainbow trout | 48 | 379 | 10 | 0.11 | 0.13 | 0.065 | 6.5 | 15 | (Freda and McDonald, 1988) |
| *Oncorhynchus mykiss* | Rainbow trout | 55 | 550 | 2.42 | 0.595 | 0.89 | 1.04 | 7.8 | 15 | (Lauren and Mcdonald, 1987) |
| *Oncorhynchus mykiss* | Rainbow trout | 69 | 683 | 4.44 | 0.05 | 0.04 | 0.02 | 5.8 | 12 | (Grosell and Wood, 2002) |
| *Oncorhynchus mykiss* | Rainbow trout | 92.7 | 351.7 | 400 | 0.6 | 0.8 | 1 | 8 | 13 | (Salama *et al.*, 1999) |
| *Oncorhynchus mykiss* | Rainbow trout | 100 | 450 | 325 | 0.6 | 0.8 | 1 | 8 | 15 | (Goss and Wood, 1990) |
| *Oncorhynchus mykiss* | Rainbow trout | 111.6 | 378.3 | 400 | 0.6 | 0.8 | 1 | 8 | 13 | (Salama *et al.*, 1999) |
| *Oncorhynchus mykiss* | Rainbow trout | 138 | 560 | 15.5 | 0.6 | 0.8 | 1 | 7.8 | 15 | (Postlethwaite and McDonald, 1995) |
| *Oncorhynchus mykiss* | Rainbow trout | 161 | 605 | 2.5 | 0.6 | 0.7 | 1 | 7.8 | 12 | (Matsuo *et al.*, 2004) |
| *Oncorhynchus mykiss* | Rainbow trout | 204 | 1453 | 2.5 | 0.068 | 0.07 | 0.092 | 6.8 | 12 | (Matsuo *et al.*, 2004) |
| *Oncorhynchus mykiss* | Rainbow trout | 257.1 | 613 | 250 | 0.5 | 0.7 | 1 | 7.9 | 13.5 | (Morgan *et al.*, 1997) |
| *Cleithracara maronii* | Keyhole cichlid | 49.4 | 452 | 2 | 0.1 | 0.3 | 0.1 | 7.5 | 26 | (Gonzalez *et al.*, 2021) |
| *Cleithracara maronii* | Keyhole cichlid | 429.8 | 871 | 2 | 1 | 1.2 | 0.1 | 7.5 | 26 | (Gonzalez *et al.*, 2021) |
| *Geophagus* sp | Geophagus | 111.8 | 1154.5 | 2.91 | 0.0296 | 0.0228 | 0.0094 | 6 | 25 | (Gonzalez *et al.*, 2002) |
| *Geophagus* sp | Tapajos cichlids | 56.3 | 1205 | 5.3 | 0.1 | 0.3 | 0.1 | 7.5 | 26 | (Gonzalez *et al.*, 2021) |
| *Geophagus* sp | Tapajos cichlids | 64.5 | 132 | 5.3 | 1 | 1.2 | 0.1 | 7.5 | 26 | (Gonzalez *et al.*, 2021) |
| *Danio rerio* | Zebrafish | 64 | 798 | 0.34 | 0.67 | NA | 0.95 | 5 | 26.5 | (Al-Reasi *et al.*, 2016) |
| *Danio rerio* | Zebrafish | 74 | 1160 | 0.39 | 0.035 | 0.043 | 0.0044 | 6 | 26.5 | (Boisen *et al.*, 2003) |
| *Danio rerio* | Zebrafish | 112 | 835 | 0.44 | 0.8 | 0.5 | 0.25 | 8 | 28 | (Kumai *et al.*, 2011) |
| *Danio rerio* | Zebrafish | 160 | 525 | 0.39 | 1.48 | 1.625 | 3.246 | 8.15 | 26.5 | (Boisen *et al.*, 2003) |
| *Danio rerio* | Zebrafish | 246 | 859 | 0.34 | 0.67 | NA | 0.95 | 7.75 | 26.5 | (Al-Reasi *et al.*, 2016) |
| *Hyphessobrycon rosaceus* | Rosy tetra | 64.1 | 1663 | 1.324 | 0.1 | 0.3 | 0.1 | 7.5 | 26 | (Gonzalez *et al.*, 2018) |
| *Hyphessobrycon rosaceus* | Rosy tetra | 65.6 | 1099 | 1.324 | 1 | 1.2 | 0.1 | 7.5 | 26 | (Gonzalez *et al.*, 2018) |
| *Pterophyllum scalare* | Angelfish | 79.3 | 533.6 | 3.39 | 0.031 | 0.049 | 0.009 | 6 | 29 | (Duarte *et al.*, 2013) |
| *Pterophyllum scalare* | Angelfish | 136.1 | 428 | 2.3 | 0.05 | 0.2 | 0.05 | 6.5 | 26 | (Gonzalez and Wilson, 2001) |
| *Apistogramma macmasteri* | Macmaster's dwarf cichlid | 108.9 | 619 | 1.6 | 1 | 1.2 | 0.1 | 7.5 | 26 | (Gonzalez *et al.*, 2021) |
| *Apistogramma macmasteri* | Macmaster's dwarf cichlid | 142.9 | 473 | 1.6 | 0.1 | 0.3 | 0.1 | 7.5 | 26 | (Gonzalez *et al.*, 2021) |
| *Astronotus ocellatus* | Oscar | 123.8 | 1165 | 5.6 | 0.1 | 0.3 | 0.1 | 7.5 | 26 | (Gonzalez *et al.*, 2021) |
| *Astronotus ocellatus* | Oscar | 201.3 | 641 | 5.6 | 1 | 1.2 | 0.1 | 7.5 | 26 | (Gonzalez *et al.*, 2021) |
| *Enneacanthus obesus* | Banded sunfish | 125 | 128 | 9.5 | 0.044 | 0.075 | 0.025 | 5.8 | NA | (Gonzalez and Dunson, 1989) |
| *Corydoras julii* | Julii cory | 147.8 | 3604.6 | 1.45 | 0.0188 | 0.0214 | 0.0088 | 6.5 | 25 | (Gonzalez *et al.*, 2002) |
| *Notropis cornutus* | Common shiner | 158 | 460 | 10 | 0.11 | 0.13 | 0.065 | 6.5 | 15 | (Freda and McDonald, 1988) |
| *Apistogramma* sp | Apistogramma | 258.5 | 1752.5 | 1.19 | 0.0188 | 0.0214 | 0.0088 | 6.5 | 25 | (Gonzalez *et al.*, 2002) |
| *Symphysodon discus* | Red discus | 267.6 | 537.9 | 40.47 | 0.031 | 0.049 | 0.009 | 6 | 29 | (Duarte *et al.*, 2013) |
| *Satanoperca jurupari* | Demon eartheater | 276.7 | 457.1 | 9.9 | 0.0188 | 0.0214 | 0.0088 | 6.5 | 25 | (Gonzalez *et al.*, 2002) |

**References**

Al-Reasi HA, Smith SD, Wood CM (2016) The influence of dissolved organic matter (DOM) on sodium regulation and nitrogenous waste excretion in the zebrafish (*Danio rerio*). *J Exp Biol* 219: 2289–2299.

Boisen AMZ, Amstrup J, Novak I, Grosell M (2003) Sodium and chloride transport in soft water and hard water acclimated zebrafish (*Danio rerio*). *Biochim Biophys Acta Biomembr* 1618: 207–218.

Duarte RM, Ferreira MS, Wood CM, Val AL (2013) Effect of low pH exposure on Na^+^ regulation in two cichlid fish species of the Amazon. *Comp Biochem Physiol A* 166: 441–448.

Freda J, McDonald DG (1988) Physiological correlates of interspecific variation in acid tolerance in fish. *J Exp Biol* 136: 243–258.

Glover CN, Donovan KA, Hill J V. (2012) Is the habitation of acidic-water sanctuaries by galaxiid fish facilitated by natural organic matter modification of sodium metabolism? *Physiol Biochem Zool* 85: 460–469.

Gonzalez RJ, Cradeur A, Guinnip M, Mitchell A, Reduta V (2018) South American characids share very similar ionoregulatory characteristics. *Comp Biochem Physiol A* 226: 17–21.

Gonzalez RJ, Dalton VM, Patrick ML (1997) Ion regulation in ion-poor acidic water by the blackskirt tetra (*Gymnocorymbus ternetzi*), a fish native to the amazon river. *Physiol Zool* 70: 428–435.

Gonzalez RJ, Dunson WA (1989) Differences in low pH tolerance among closely related sunfish of the genus *Enneacanthus*. *Environ Biol Fishes* 26: 303–310.

Gonzalez RJ, Hsu R, Mahaffey L, Rebagliatti D, Shami J (2021) Examination of ionoregulatory characteristics of South American cichlids. *Comp Biochem Physiol A* 253: 110854.

Gonzalez RJ, Jones SL, Nguyen T V. (2017) Ionoregulatory characteristics of non–Rio Negro characiforms and cichlids. *Physiol Biochem Zool* 90: 407–414.

Gonzalez RJ, Preest MR (1999) Ionoregulatory specializations for exceptional tolerance of ion-poor, acidic waters in the neon tetra (*Paracheirodon innesi*). *Physiol Biochem Zool* 72: 156–163.

Gonzalez RJ, Wilson RW (2001) Patterns of ion regulation in acidophilic fish native to the ion-poor, acidic Rio Negro. *J Fish Biol* 58: 1680–1690.

Gonzalez RJ, Wilson RW, Wood CM, Patrick ML, Val AL (2002) Diverse strategies for ion regulation in fish collected from the ion-poor, acidic Rio Negro. *Physiol Biochem Zool* 75: 37–47.

Goss GG, Wood CM (1990) Na^+^ and Cl^-^ uptake kinetics, diffusive effluxes and acidic equivalent fluxes across the fills of rainbow trout I. Response to environmental hyperoxia. *J Exp Biol* 152: 521–547.

Grosell M, Wood CM (2002) Copper uptake across rainbow trout gills: mechanisms of apical entry. *J Exp Biol* 205: 1179–1188.

Kumai Y, Bahubeshi A, Steele S, Perry SF (2011) Strategies for maintaining Na^+^ balance in zebrafish (*Danio rerio*) during prolonged exposure to acidic water. *Comp Biochem Physiol A* 160: 52–62.

Lauren DJ, Mcdonald DG (1987) Acclimation to copper by rainbow trout, *Salmo gairdneri*: physiology. *Can J Aquat Sci* 44: 99–104.

Matsuo AYO, Playle RC, Val AL, Wood CM (2004) Physiological action of dissolved organic matter in rainbow trout in the presence and absence of copper: Sodium uptake kinetics and unidirectional flux rates in hard and softwater. *Aquat Toxicol* 70: 63–81.

Matsuo AYO, Val AL (2007) Acclimation to humic substances prevents whole body sodium loss and stimulates branchial calcium uptake capacity in cardinal tetras *Paracheirodon axelrodi* (Schultz) subjected to extremely low pH. *J Fish Biol* 70: 989–1000.

Morgan IJ, Henry RP, Wood CM (1997) The mechanism of acute silver nitrate toxicity in freshwater rainbow trout (*Oncorhynchus mykiss*) in inhibition of gill Na^+^ and Cl^-^ transport. *Aquat Toxicol* 38: 145–163.

Postlethwaite EK, McDonald DG (1995) Mechanisms of Na^+^ and Cl^-^ regulation in freshwater-adapted rainbow trout (*Oncorhynchus mykiss*) during exercise and stress. *J Exp Biol* 198: 295–304.

Preest MR, Gonzalez RJ, Wilson RW (2005) A pharmacological examination of Na^+^ and Cl^-^ transport in two species of freshwater fish. *Physiol Biochem Zool* 78: 259–272.

Salama A, Morgan IJ, Wood CM (1999) The linkage between Na^+^ uptake and ammonia excretion in rainbow trout: kinetic analysis, the effects of (NH_4_)_2_SO_4_ and NH_4_HCO_3_ infusion and the influence of gill boundary layer pH. *J Exp Biol* 202: 697–709.

Shartau RB, Brix K V., Brauner CJ (2017) Characterization of Na^+^ transport to gain insight into the mechanism of acid-base and ion regulation in white sturgeon (*Acipenser transmontanus*). *Comp Biochem Physiol A* 204: 197–204.
